# Working from home and health complaints: on the difference between telework and informal overtime at home

**DOI:** 10.3389/fpubh.2025.1465617

**Published:** 2025-02-18

**Authors:** Alexandra Mergener, Nico Stawarz, Heiko Rüger, Inga Laß

**Affiliations:** ^1^Federal Institute for Vocational Education and Training (BIBB), Bonn, Germany; ^2^Federal Institute for Population Research (BiB), Wiesbaden, Germany; ^3^Melbourne Institute of Applied Economic and Social Research, The University of Melbourne, Carlton, VIC, Australia

**Keywords:** remote work, mental health, physical health, musculoskeletal complaints, job demands, job resources, Germany

## Abstract

**Background:**

With the increase in the prevalence of working from home (WFH), understanding its impact on health has become more relevant. However, the possibility that health effects may depend on the specific WFH arrangement has largely been ignored in research.

**Objective:**

The aim of this study is to offer a differentiated view of WFH by distinguishing between informal overtime at home and telework during recognized working time when assessing its associations with mental and physical health complaints. Moreover, the extent of telework is considered. The study also differentiates the group of WFH non-users by distinguishing between voluntary non-use and employer-directed non-use.

**Methods:**

We apply OLS regression models with clustered standard errors by occupation to probability-based survey data that is representative of employees in Germany. The analytical sample was restricted to employees whose job tasks could be performed at home (*N* = 10,365).

**Results:**

Compared to employer-directed non-users, working informal overtime at home is associated with more mental health complaints, while telework is associated with fewer mental and physical health complaints. However, the beneficial association between recognized telework and mental health only applies to employees with relatively small extents of telework. At higher extents of telework, the mental health advantages disappear, while those for physical health tend to remain.

**Conclusion:**

This study suggests that a nuanced look at patterns of use and non-use of WFH is essential when gauging its impact on health.

## Introduction

One of the major changes affecting workplace organization is the rise in the prevalence of working from home (WFH), both in the wake of the COVID-19 pandemic and as a result of a general trend toward widespread use of information and communication technologies in the digital age. WFH refers to a work practice where employees perform job-related tasks for the employer at least partially from home. It is often argued that this work pattern may positively affect workers’ physical and mental health [e.g., (1)]. It might allow more flexibility in terms of working time and place, more autonomous work organization, a reduction in the need for commuting, and more time for family and leisure (2–4), all of which can be considered job resources and promote employees’ health [e.g., (5)]. However, a decisive factor in the impact on health appears to be how WFH arrangements are organized and supported by the employer (1). When leading to overtime, work intensification, stress, social isolation, or when ergonomic work equipment and a dedicated working area are not available, WFH can also constitute a job demand that creates health risks.

Against this background, this paper explores the relationship between WFH and both mental and physical health. Specifically, it asks (1) whether WFH is associated with fewer mental and physical health complaints compared to employer-directed non-WFH use, (2) how the associations differ between employees doing informal overtime at home and employees doing telework within recognized working hours, and (3) whether associations differ with the extent of telework. A deeper understanding of these issues is of high societal relevance. Mental and behavioral conditions lead the ranking in terms of disease expenditure in Germany (13.1% of the total €432 billion in 2020), and musculoskeletal conditions account for another 9.6% [own calculations based on (61)]. Musculoskeletal conditions are also the most frequent cause of sickness absences (6). Furthermore, health problems can have repercussions for a range of other areas of life. For example, major depressive disorders have been associated with low marital quality, reduced work performance and earnings, various chronic physical disorders, and even early mortality (7).

Literature reviews elaborate on benefits and drawbacks of WFH [e.g., (8–10)] and point to an overall health-promoting effect of WFH. However, they also highlight potential negative health aspects of WFH and, more importantly, reveal substantial knowledge gaps that need to be closed in order to adequately assess the relationship. *First*, persons who do and do not engage in WFH are often considered as uniform groups, although employees differ in the possibilities, motivations and extent of WFH. In this regard, Koh et al. (11) point out that ignoring the difference between self-decided non-use of WFH and lack of employer permission might introduce bias. Moreover, WFH can either be based on a formal agreement with the employer or, in contrast, can be used to catch up on work at home, mostly as informal overtime, e.g., in the evenings and/or on weekends [e.g., (12, 13)]. The reasons and motivations for the two forms differ (14) and may range from reducing commuting time or better balancing personal and work commitments to meeting deadlines or career aspirations, suggesting different relationships with health. In addition, the relationship may depend on the extent of WFH. For example, in their current meta-analyses, Beckel et al. (15) refer to different results on the effect of WFH on work–family conflict, depending on whether WFH is measured as a continuous or dichotomous variable. However, the role of the extent of WFH has been under-researched in the examination of the relationship between WFH and health (8). *Second*, most studies (can) consider only a global indicator of general health, while research analyzing the effects of WFH on mental and physical health separately is scarce (10). However, in evaluating the health risks and benefits of WFH to inform policy and enrich public health debates, it is important to consider whether mental and/or physical health is affected, as the advice given and measures taken may differ. *Third*, most research in the field of WFH and health consists of case studies and special (mostly small) samples (16). However, in order to generalize the findings to the population of employees, representative and large-scale data are needed.

To address these research gaps, we examine the relationship between WFH and health in a systematic and differentiated way. We explicitly decompose WFH into *informal overtime at home* and *telework.* Informal overtime at home means additional hours of work that are usually not recognized by the employer, e.g., in the evening after a full day in the office. According to Allen et al. (8), telework means a work practice that enables employees to substitute (at least part of) their regular work hours, mostly spent in the employer’s premises, with recognized hours worked from home. When considering telework, we also take the extent into account. In addition, we aim to avoid possible confounding bias in the group of non-users of WFH by considering non-use due to job tasks, lack of permission from the employer, or the employee’s own choice. Finally, we contribute to the literature by assessing the association of WFH patterns with both self-reported mental and physical complaints. For this purpose, we use data from German employees from the 2018 Employment Survey of the Federal Institute for Vocational Education and Training (BIBB) and the Federal Institute for Occupational Safety and Health (BAuA) (17). The BIBB/BAuA Employment Survey is a probability-based, large-scale survey representative of employees in Germany. We consider the fact that this survey was conducted before the COVID-19 pandemic as a strength since it removes potential bias due to infection control measures [e.g., (18)], such as politically mandated home-based work, shutdowns and temporary closures of childcare facilities and schools. WFH during the exceptional pandemic situation may have had a particular impact on health-related outcomes that cannot be generalized to post-COVID WFH patterns.

## Conceptual framework

The potential effects of WFH on subjective health can be contextualized within the theoretical model of job demands and resources (JD-R) (19, 20). The JD-R model defines job demands, such as time pressure or high workload, as factors that require physical and/or mental effort, whereby high demands can exhaust existing resources and can be related to strain and lower subjective health. In contrast, job resources, such as autonomy or time flexibility can reduce physiological and/or mental costs related to job demands and thus buffer the effect of job demands on job strain, thereby potentially improving subjective health.

In this theoretical context, WFH in the sense of a telework arrangement is often defined as a job resource, because it increases, e.g., time and spatial flexibility as well as autonomy (13, 21, 22). However, WFH can also be seen as a job demand, especially when occurring as informal overtime at home, as it can be linked to work intensification and increased work to non-work conflict through role blurring (13, 23, 24). Ultimately, how and to what extent employees perform WFH and whether it is a job resource or demand depends on their reasons for WFH.

### Employees’ reasons for (not) using WFH

Mokhtarian and Salomon (25) argue that the decision for WFH is determined by facilitators, constraints, and drives. Facilitators are factors that allow changes into WFH, like managerial approval, appropriate technological or spatial conditions. By contrast, constraints hinder the uptake of WFH, e.g., the lack of a workspace in the home, lack of self-discipline on the part of the employee, or job-related constraints. Perceived job unsuitability and presence in the workplace expected by the supervisor have been reported as the major constraints in the uptake of WFH (26). Finally, drives are factors that motivate individuals to consider WFH. These drives can arise from the spheres of work, family, leisure, or commuting, such as the wish to improve the compatibility of family and work obligations as well as career reasons or to work overtime.

From these basic assumptions, we can derive different scenarios related to WFH use. First, when facilitators outweigh or equal constraints and a sufficient drive exists, employees will consider starting WFH. Second, when facilitators outweigh or equal constraints but the drive to consider WFH is low or lacking, workers will usually not adopt WFH. Third, when a sufficient drive exists but the constraints outweigh the facilitators, workers will also not use WFH.

These scenarios illustrate the heterogeneity in both the groups of users and non-users of WFH, which has to be considered when evaluating associations with subjective health. Where WFH is restricted by the employer but a sufficient drive on the part of employees exists, employees most probably see WFH as a resource for coping with existing job demands, e.g., to increase their flexibility or to improve the compatibility of private and work obligations. This employee group would very likely take up WFH, e.g., when the employers’ preference changes, which could then positively impact their subjective health. We see this group of WFH non-users as appropriate counterfactuals to current WFH users, because they are comparable in their motivations but unable to use WFH. In the following, we refer to them as “employer-directed non-WFH users,” which is the base category (i.e., the comparison group) for the following hypothesis tests. In contrast, where WFH is possible but no sufficient drive exists, we do not expect employees to gain health benefits from WFH. Thus, a take-up of WFH is unlikely under current conditions. We name them “voluntary non-WFH users.” Finally, the situation where WFH is used needs to be further distinguished into informal overtime at home and telework (13, 27). WFH as informal overtime at home, which is performed in a more unregulated manner outside formally recognized working hours, should more likely be due to reasons like career aspirations or meeting deadlines. In contrast, when teleworking, persons use WFH in a regulated manner within recognized working hours, which should more probably be rooted in reasons like working undisturbed or more efficient, reduced commuting, or increased compatibility of private and work obligations.

### Informal overtime at home and employees’ health

For informal overtime at home, we generally assume that it is likely to represent a job demand (20), which ultimately leads to higher strain and reduced subjective health. The literature suggests that informal overtime at home is related to negative emotions (27), work-to-family conflict (24, 28), less free time, and less time for sports (29, 30). Furthermore, it can be assumed that employees who only work overtime at home and do not formally telework do not have an adequately equipped workplace. There is therefore a risk of incongruous posture. Moreover, as commuting is not reduced, but even more frequent during peak hours (31), there is also no stress reduction (30). Therefore, research suggests that informal overtime at home may be associated with emotional exhaustion and psychological distress, low job satisfaction (32), sleep problems (28), risk of burnout (33), and ultimately reduced well-being and health (24, 29). When examining how WFH is related to employees’ subjective health, it therefore appears important to separate informal overtime at home from telework. We put forward our first hypothesis:

*H1:* Informal overtime at home is positively associated with subjective (a) mental and (b) physical health complaints compared to employer-directed non-WFH users.

### Telework and employees’ health

Telework, in contrast, has a greater potential to be a resource for employees. Advantages of telework are autonomy in the organization of own work, flexibility to coordinate personal and professional life as needed, and less psychological job demands (34). This reduces stress and work-to-family conflict, strengthen a sense of self-determination and, thus, positively affect aspects of mental and physical health [e.g., (2, 22, 35)] and subjective overall health (36). Moreover, it can allow working undisturbed and without interruptions, can lead to reductions in commuting and to more commitment to the employer, which also have positive impacts on mental and physical health [e.g., (5, 37, 38)]. Since employees likely decide to telework primarily to take advantage of these benefits, we assume that:

*H2*: Telework is negatively associated with subjective (a) mental and (b) physical health complaints compared to employer-directed non-WFH users.

Literature reviews, however, also discuss several negative factors of telework in relation to health [e.g., (4, 9, 39)]. On the downsides of telework are blurred boundaries between work and private life, a reduction of restorative effects of being at home, isolation and loneliness, lower social support by colleagues, work intensification and inadequate work equipment or ergonomic issues that should be negatively related to mental and physical health [e.g., (5, 40, 41)]. Whether the positive or negative mechanisms predominate strongly depends on the *extent* of telework (4, 8).

### The role of the extent of telework

On the one hand, when employees telework to a very small extent or irregularly, it is more likely occasion-based, so that private appointments, e.g., with the doctor or craftsmen, and family obligations can be integrated into the workday. In this case, the benefits of telework, particularly the higher flexibility and autonomy, should dominate over the drawbacks, such as isolation and loneliness or inadequate work equipment, leading to a reduction in both mental and physical health complaints.

On the other hand, if telework is used to a larger extent and thus more regularly, we assume that the upsides and downsides are more likely to cancel each other out with respect to mental health. Frequent teleworkers tend to live farther away from the employer’s premises and/or have a high need to integrate personal demands into their daily work routine. Here, a reduction of mental distress can be expected due to the time saved by not having to commute and less work–family conflict when teleworking frequently (3). In addition, disturbances and interruptions from co-workers should also decrease. Regarding the job resources of autonomy and work-time control, which can also lead to reduced mental stress, existing studies are, however, inconclusive whether or not these increase with the extent of telework [e.g., (2, 5, 22, 35)]. However, there is much evidence that social integration and relationships with colleagues can suffer from large extents of telework, which can negatively impact mental health. Ultimately, we assume that:

*H3a*: Only a small extent of telework is negatively associated with subjective mental health complaints compared to employer-directed non-WFH users.

In contrast, the benefits for physical health may increase further with larger extents of teleworking. The relieving effects of less commuting^1^ on physical health, e.g., due to more time for sports and leisure as well as better eating habits, should increase with the extent of telework (8, 42–44). Additionally, we expect that negative effects of poor workplace conditions at home, like inadequate work equipment, visual overload or ergonomic issues, decrease with the extent of telework (41, 45), since employees working frequently at home should have better equipped workplaces or optimize their workplaces gradually. In Germany, e.g., contractual telework arrangements require employers to provide office equipment and conduct a health risk assessment of teleworkers’ working conditions at home. Thus, we assume that the extent of telework will be negatively associated with physical health complaints. We thus put forward our last hypothesis:

*H3b*: Higher telework extents are negatively associated with physical health complaints compared to employer-directed non-WFH users.

## Materials and methods

### Data and sample

The study draws on data from the German BIBB/BAuA Employment Survey 2018 (46), a probability-based large-scale survey representative of German-speaking individuals working at least 10 h per week. The data comprise detailed measures of WFH and subjective health as well as a large number of socio-demographic and job-related characteristics, which enables the adjustment for relevant control variables to avoid possible confounding bias.

Given that our focus is on the decomposition of WFH use, specifically whether the employer recognizes the working hours at home (telework) or not (informal overtime), and WFH non-use, specifically considering employer-directed non-WFH users as counterfactuals, we restrict our sample to employees. More precisely, we restricted it to employees aged 18–65 years reporting that their job tasks can theoretically be done from home at least partly or temporarily to form a homogenous group of (potential) WFH users. The final analytical sample includes only those with complete information on the outcomes, predictor variable, and potential confounders (*N* = 10,365).

### Measures

#### Dependent variables: subjective mental and physical health complaints

Following BAuA (47), we built separate indices for mental complaints and for physical complaints to operationalize the subjective health of employees. For mental complaints, participants indicated yes or no to whether nervousness or irritability, insomnia, fatigue or exhaustion, and dejection had occurred frequently on workdays during the last 12 months. Physical complaints referred to the frequent occurrence of different types of musculoskeletal pain, i.e., in the back, neck or shoulders, arms, hands, hips, legs or feet, knees, and swollen legs. Each index represented the number of positive responses (with no missing information allowed on any of the variables) as a share of the total number of possible complaints (in percent). The internal consistency (Cronbach’s Alpha) in the present sample was *α* = 0.75 for the mental complaints index and α = 0.71 for the physical complaints index.

#### Key predictor variable: WFH

WFH was measured by four different questions: the usage of WFH, the recognition of home working hours, whether the employer allows it, and the extent of WFH. First, employees were asked whether or not they worked from home for the employer at least partly or temporarily. Employees who indicated that they did not work from home could then answer the question “If your employer allowed you to work from home from time to time, would you do so?.” Those who responded that they would do so if their employer allowed it, were classified as *employer-directed non-WFH* users, while employees who would not work from home even if their employer allowed it were classified as *voluntary non-WFH* users. Employees who work from home were also asked “Are the hours you work from home recognized as working time by your employer?” Those responding with yes were classified as *teleworkers*. Employees who indicated that they work from home without recognition of home-working hours were classified as doing *informal overtime at home*.

Additionally, respondents who worked from home were asked “How many hours a week do you usually work from home on average?,” and could give the exact number of hours. Individuals who rarely use WFH and did not specify an exact number could choose the category “irregular.” For employees using telework, we created a measure of the share of time worked from home by dividing the number of hours usually worked from home per week by the total number of weekly working hours in their job. We categorized the share of WFH into three groups: < 20% or irregular, 21–80%, and > 80% or always. This categorization also allows us to identify possible non-linear relationships. Note that a further differentiation of the relatively broad middle category was not possible due to relatively few employees being in that category.

#### Confounders

We controlled for a rich set of individual-level, job, company and regional variables that can be determinants of both WFH and health [e.g., (48–50)]. In terms of individual-level variables, we considered sex, age, educational level, whether employees had a disability, and whether they lived with a partner and/or children in the household.

In terms of job and company characteristics, we controlled for employees’ wage, managerial position and total weekly working hours. To disentangle the effects of informal overtime at home and in the employer’s premises, we also considered overtime at the company as a categorical variable, where we distinguished between no overtime, compensated overtime, and unpaid overtime.^2^ We also accounted for physical effort at work using an index based on how often employees have to lift heavy loads, stand, use their hands, and stoop (ranging from 0 “never” to 3 “often”), as WFH is more likely to be used by employees in jobs with low physical effort (51). Furthermore, we controlled for company size and the presence of a works council.

Finally, we accounted for the rurality of employees’ place of residence. The rurality indicator was matched from official statistics of the German Federal Institute for Research on Building, Urban Affairs and Spatial Development, see BBSR (52). It describes the proportion of inhabitants in municipalities with a population density of less than 150 inhabitants/km^2^ and indicates rural dispersed settlement structures. Access to medical care and also to technical possibilities, i.e., broadband coverage—often a prerequisite for WFH—still differs significantly between rural and urban regions (53).

Summary statistics of all variables are presented in Table 1. The mean score for the mental health index is 32.5, which is considerably higher than the mean score for physical complaints (20.6). Among our sample, 31% were employer-directed non-WFH users, about 15% were voluntary non-WFH users, 10% were doing informal overtime at home, and close to 45% engaged in telework. Considering the extent of telework, we found that 27% of employees worked from home for a relatively small share of their total working time, i.e., less than 20% or irregularly. Furthermore, 8% worked from home between 21 and 80% of their total working time and about 9% teleworked more than 80% of their time. A closer look at how the groups of WFH non-users and users differ in key work and individual characteristics shows that, compared to the reference category of employer-directed non-WFH users, teleworkers are more likely to be highly educated, to have a high income, to work full-time and in companies with work councils, to live with a partner in the household and to have children. Employees doing informal overtime at home are more likely to be in middle or upper management than employer-directed non-WFH users.

**Table 1:** Distribution of sample characteristics (categorical variables in percent, continuous variables with mean and standard deviations in parentheses).

		WFH (non)-user groups					
		Employer-directed non-WFH use	Voluntary non-WFH use	Informal overtime at home	Telework		
					< 20%, irregular	21–80%	> 80%, always
	Total	31.1	14.5	9.9	27.4	8.4	8.8
Mental complaints	32.45 (34.4)	34.46 (35.4)	27.83 (32.8)	39.37 (35.5)	29.00 (32.9)	31.80 (33.7)	36.61 (34.8)
Physical complaints	20.60 (21.6)	24.16 (23.6)	21.47 (22.6)	21.29 (21.4)	17.39 (19.2)	17.45 (18.8)	18.81 (19.9)
Women (Reference: men)	51.9	56.6	60.6	44.1	44.3	47.9	57.9
Age groups							
18–35 years	20.0	23.1	16.9	17.3	21.0	17.8	16.5
36–45 years	22.6	22.2	19.9	23.5	24.4	24.7	19.5
46–55 years	33.7	33.8	33.7	31.3	34.0	35.7	33.6
56–65 years	23.7	20.9	29.5	27.9	20.6	21.8	30.5
Disability (Reference: no disability)	9.3	10.9	11.7	9.3	7.3	7.5	7.7
Partner living in household (Reference: no partner living in household)	69.6	66.2	63.5	72.8	72.6	76.2	72.0
Children							
No children in household	64.9	66.0	72.4	64.9	62.0	57.4	65.0
Children aged <6 years	12.2	12.0	7.7	13.1	13.6	15.8	12.0
Children aged 6 or more	22.8	22.0	19.9	22.1	24.4	26.8	23.0
Educational level							
No vocational degree	3.2	4.3	5.7	1.6	2.1	2.2	1.5
Vocational education	39.0	55.4	53.3	31.0	29.3	19.2	15.5
Further vocational training	8.7	10.6	7.6	9.1	9.2	4.8	5.8
Academic degree	49.1	29.7	33.5	58.3	59.4	73.8	77.1
Physical effort	0.97 (0.8)	1.03 (0.9)	1.03 (0.9)	1.12 (0.9)	0.85 (0.8)	0.76 (0.7)	1.07 (0.7)
Management position							
No management position	67.3	72.7	73.1	48.6	61.1	70.0	76.4
Lower management	8.9	9.7	7.5	8.8	10.4	7.9	5.2
Middle management	17.3	14.6	15.9	27.0	20.0	14.0	12.5
Upper management	6.5	3.1	3.5	15.6	8.5	8.0	5.9
Gross wage							
<2,000 euros per month	15.2	21.2	23.3	10.5	9.6	9.1	8.8
2,000–3,000 euros per month	21.3	29.5	27.7	16.9	15.9	11.9	11.9
3,001–4,500 euros per month	33.0	35.2	32.1	31.2	30.9	30.5	38.0
> 4,500 euros per month	30.6	14.1	17.0	41.4	43.7	48.5	41.4
Total weekly working time							
<20 hours	3.7	4.1	5.0	2.2	2.6	4.6	4.1
20–35 hours	19.4	22.0	26.0	14.3	16.2	17.7	16.6
≥35 hours	76.9	73.9	69.0	83.5	81.2	77.7	79.3
Overtime at the company							
No overtime in the company	41.4	47.9	54.2	41.4	32.6	37.4	28.7
Compensated overtime in the company	42.1	45.9	39.9	28.4	51.6	31.3	28.1
Unpaid overtime in the company	13.4	5.1	4.7	25.5	12.7	27.2	33.0
No info about compensation when overtime in the company	3.1	1.1	1.3	4.8	3.1	4.0	10.2
Company size							
1–9 employees	6.4	6.7	8.4	6.5	6.5	4.5	4.0
10–49 employees	23.9	25.4	23.4	24.0	21.5	20.4	29.8
50–249 employees	29.2	29.0	26.7	32.3	23.6	31.7	45.5
250 or more employees	40.5	38.9	41.5	37.2	48.4	43.4	20.7
Works council (Reference: no works council)	70.0	66.1	69.9	69.6	69.8	77.6	78.0
Rurality scale of employees’ place of residence	19.91 (16.7)	20.80 (16.6)	20.95 (16.8)	20.52 (17.3)	18.44 (16.3)	18.11 (16.4)	20.65 (17.0)
N	10,365	3,220	1,503	1,025	2,837	871	909

### Methods

We used OLS regression models for the continuous outcome variables of mental and physical complaints.^3^ We accounted for the clustering of WFH into occupations by using clustered standard errors at the occupational level (specified at the 2-digit level of the German Classification of Occupations, KldB 2010), thus achieving higher estimator precision (54).

In a first step, we included WFH as a dummy variable that distinguishes between employees who do and do not work from home, as is used in many other studies. Second, we further differentiated the group of WFH non-users into employer-directed non-WFH users (reference category) and voluntary non-WFH users, as well as the group of WFH users into doing informal overtime at home and teleworkers. As argued above, employer-directed non-WFH users are appropriate counterfactuals because they should be more similar in their motives to current WFH users than voluntary non-WFH users. Third, we accounted for the extent of telework.

## Results

To gain insights into the association between WFH and health, OLS regression models were estimated. Table 2 shows the results for employees’ mental and physical complaints by WFH patterns, including all control variables. In M1a and M1b, we started with considering WFH as a dichotomous variable. Our findings revealed that employees who did any work from home had statistically significantly more mental complaints but less physical complaints than employees who did not work from home. Precisely, mental complaints were elevated by 2.3 percentage points, and physical complaints reduced by 1.2 percentage points.

**Table 2:** Association between WFH and mental and physical complaints.

	(M1a)	(M1b)	(M2a)	(M2b)	(M3a)	(M3b)
	Mental complaints	Physical complaints	Mental complaints	Physical complaints	Mental complaints	Physical complaints
No WFH	Reference	Reference				
Employer-directed non-WFH use			Reference	Reference	Reference	Reference
Voluntary non-WFH use			−6.766***	−3.331***	−6.741***	−3.333***
			(1.021)	(0.769)	(1.025)	(0.772)
WFH	2.297**	−1.168*				
	(0.757)	(0.468)				
Informal overtime at home			5.642***	−0.727	6.240***	−0.959
			(1.022)	(0.607)	(1.183)	(0.578)
Telework			−1.380	−2.649***		
			(1.051)	(0.638)		
<20%, irregular					−2.411**	−2.555***
					(0.865)	(0.645)
21–80%					0.683	−1.565
					(1.397)	(0.952)
> 80%, always					1.625	−3.422***
					(2.001)	(0.890)
Confounders						
Women (Reference: men)	7.512***	7.596***	7.613***	7.636***	7.484***	7.669***
	(0.698)	(0.580)	(0.710)	(0.570)	(0.735)	(0.568)
Age (Reference: 18–35 years)						
36–45 years	2.309*	2.803***	2.415*	2.867***	2.367*	2.861***
	(0.973)	(0.604)	(0.946)	(0.604)	(0.942)	(0.604)
46–55 years	2.866***	5.167***	3.039***	5.250***	2.962***	5.252***
	(0.774)	(0.647)	(0.780)	(0.656)	(0.768)	(0.653)
56–65 years	3.072**	6.778***	3.297**	6.917***	3.146**	6.951***
	(0.971)	(0.715)	(0.967)	(0.733)	(0.954)	(0.728)
Disability (Reference: no)	10.232***	10.529***	10.095***	10.483***	10.080***	10.490***
	(1.134)	(0.739)	(1.164)	(0.767)	(1.157)	(0.769)
Partner living in household (Reference: not)	−3.440***	0.074	−3.477***	0.048	−3.501***	0.036
	(0.910)	(0.516)	(0.882)	(0.518)	(0.900)	(0.523)
Children in household (Reference: no)						
Children aged <6 years	−2.215*	−1.291	−2.356*	−1.377	−2.453*	−1.403
	(1.071)	(0.816)	(1.051)	(0.796)	(1.068)	(0.798)
Children aged 6 or more	−1.204	−1.527*	−1.237	−1.561*	−1.283	−1.580*
	(0.974)	(0.642)	(0.953)	(0.636)	(0.965)	(0.640)
Vocational degree (Reference: no)						
Vocational education and training	−0.580	−1.165	−1.077	−1.367	−1.038	−1.345
	(1.764)	(1.409)	(1.776)	(1.418)	(1.772)	(1.415)
Further vocational education	−1.527	−3.521*	−2.183	−3.800*	−2.206	−3.748*
	(2.046)	(1.513)	(1.964)	(1.517)	(1.949)	(1.509)
Academic degree	−2.999	−5.430***	−3.228	−5.537***	−3.471	−5.511***
	(1.942)	(1.377)	(1.896)	(1.378)	(1.807)	(1.377)
						
Physical effort	5.955***	6.864***	5.827***	6.837***	5.723***	6.879***
	(0.542)	(0.577)	(0.552)	(0.575)	(0.547)	(0.577)
Management position (Reference: no management responsibility)						
Lower management	0.305	0.214	0.006	0.104	0.281	0.087
	(0.747)	(0.575)	(0.733)	(0.581)	(0.699)	(0.593)
Middle management	−2.494*	−0.994	−2.874**	−1.078	−2.615**	−1.080
	(1.027)	(0.543)	(0.996)	(0.548)	(0.932)	(0.555)
Upper management	−4.604***	−1.845*	−5.457***	−2.064*	−5.132***	−2.053*
	(1.134)	(0.768)	(1.144)	(0.780)	(1.113)	(0.774)
Gross wage (Reference: <2000 euros per month)						
2000–3,000 euros per month	3.088**	−0.794	3.144**	−0.791	3.096**	−0.796
	(1.085)	(0.836)	(1.057)	(0.841)	(1.066)	(0.842)
3,001–4,500 euros per month	2.777	−2.485*	2.996	−2.429*	2.823	−2.418*
	(2.016)	(1.077)	(2.025)	(1.084)	(2.110)	(1.089)
> 4,500 euros per month	0.134	−3.166**	0.622	−3.013*	0.437	−3.041*
	(1.764)	(1.163)	(1.744)	(1.162)	(1.790)	(1.179)
Total weekly working time (Reference: <20 hours)						
20–35 hours	9.724***	4.475***	9.590***	4.443***	9.748***	4.456***
	(2.253)	(1.041)	(2.247)	(1.054)	(2.263)	(1.048)
≥35 hours	11.793***	7.491***	11.322***	7.323***	11.494***	7.353***
	(2.150)	(1.208)	(2.077)	(1.190)	(2.091)	(1.189)
Overtime (Reference: no overtime in the company)						
Compensated overtime in the company	3.397**	0.852	3.610***	0.874	3.743***	0.874
	(1.000)	(0.487)	(0.978)	(0.505)	(0.994)	(0.509)
Unpaid overtime in the company	8.126***	1.139	7.927***	1.056	7.319***	1.111
	(1.167)	(0.780)	(0.906)	(0.746)	(1.119)	(0.747)
No info about compensation when overtime in the company	4.149	0.284	4.183	0.272	3.608	0.448
	(2.414)	(1.026)	(2.520)	(1.013)	(2.304)	(1.050)
Company size (Reference: 1–9 employees)						
10–49 employees	1.193	1.234	1.050	1.142	0.853	1.146
	(1.461)	(0.936)	(1.451)	(0.921)	(1.471)	(0.925)
50–249 employees	2.181	1.375	1.929	1.253	1.677	1.266
	(1.194)	(0.940)	(1.208)	(0.935)	(1.241)	(0.937)
250/more employees	0.474	2.074*	0.336	2.005*	0.466	1.950*
	(1.470)	(0.942)	(1.483)	(0.951)	(1.425)	(0.944)
Works council (Reference: no)	0.777	−0.575	0.866	−0.530	0.618	−0.517
	(0.691)	(0.781)	(0.676)	(0.775)	(0.726)	(0.763)
Rurality scale of employees’ place of residence	−0.015	0.004	−0.016	0.003	−0.017	0.004
	(0.022)	(0.008)	(0.022)	(0.008)	(0.022)	(0.008)
Constant	7.25**	3.438	10.171***	4.803*	10.62***	4.690*
	(2.512)	(2.380)	(2.396)	(2.239)	(2.676)	(2.238)
R^2^	0.064	0.186	0.072	0.189	0.073	0.189
Observations	10,365	10,365	10,365	10,365	10,365	10,365

B-coefficients with cluster-robust standard errors in parentheses, for *p < 0.05, ** for *p* < 0.01, *** for *p* < 0.001.

In the next step, we further differentiated both the group of WFH users and non-users. M2a and M2b show that employees using WFH only for informal overtime had more mental health complaints than employer-directed non-WFH users, confirming H1a. However, informal overtime WFH users did not differ significantly from the reference group in terms of physical health complaints, so we reject H1b. In contrast, those who teleworked during recognized working time reported significantly fewer physical health complaints compared to employer-directed non-WFH users. The coefficient for mental complaints was also negative, but small and statistically insignificant. This supported H2b, but not H2a. Additionally, voluntary non-WFH users showed both fewer mental and physical complaints than employer-directed non-WFH users.

In the final step, we accounted for the extent to which employees teleworked. In M3a and M3b, two trends emerged. First, employees who teleworked to a small extent (< 20% or irregularly) had significantly fewer mental and physical complaints than employer-directed non-WFH users. Second, employees who teleworked to a large extent (> 80%) also had significantly fewer physical complaints than employer-directed non-WFH users, whereas they did not differ significantly in their mental complaints from the reference group. These findings support our expectation in H3a that the negative association between telework and mental health complaints attenuates with increasing teleworking hours and only applies to a small extent of telework. Furthermore, they provide support for the assumption in H3b that higher telework extents are negatively associated with physical health complaints compared to employer-directed non-WFH users. In order to illustrate the differences across all WFH categories, we have also plotted the results of M3a and M3b as average probabilities of the respective complaints (see Supplementary Figure S1).

### Additional analysis

We conducted additional analyses to test the robustness of the results [see all robustness checks (RC) in Supplementary Table S3]. First, we examined to what extent results change if we vary our categorization of WFH non-users. On the one hand, we performed the analyses with the full sample of employees (*N* = 15,551) instead of restricting it to potential and actual WFH users in order to account for possible selection (RC 1.1). We therefore added a category for those who reported that their job tasks cannot be done from home. The results show that this group had significantly fewer mental and physical health complaints than employer-directed non-WFH users. On the other hand, we re-ran the analysis with the entire group of non-users as reference group, i.e., employer-directed and voluntary non-users together (RC 1.2). The results show that upon inclusion of the relatively healthy group of voluntary non-users in the reference category, the positive association between telework to a moderate (21–80%) or large extent (> 80%) and mental complaints became stronger and statistically significant, and the negative association between a small extent of telework (< 20%) and mental complaints attenuated and became statistically insignificant. For physical complaints, the negative associations also attenuated and became less statistically significant. These findings suggest that ignoring heterogeneity among WFH non-users in the reference group leads to an underestimation of the beneficial effects and an overestimation of the detrimental effects of WFH on subjective health.

Second, we additionally considered three categories of the share of informal overtime at home in employees’ total weekly working hours (<5%, 5–15, >15%). In the analyses (RC 2), we found each of these categories significantly positively related with mental complaints, but no statistically significant associations with physical complaints. Moreover, the coefficients of the categories of the extent of informal overtime did not differ significantly from each other in either model.

Third, we deployed employees’ general self-rated health as dependent variable, described on a five-point scale (poor, fair, good, very good, or excellent), in order to link our study to the many studies in the field that have used this broad indicator as outcome variable. The correlation between general health and mental complaints in our data was *r* = −0.41 (*p* < 0.001) and of general health and physical complaints *r* = −0.44 (p < 0.001). The ordered logit regression models analyzing the relationship between WFH and general health (RC 3) point in a very similar direction to our main analyses, namely that voluntary non-WFH users and employees who teleworked to a small extent enjoyed better health than employer-directed non-WFH users. No statistically significant association was found between informal overtime at home and general health, which is comparable to the results for physical complaints but not to those for mental complaints. This suggests that considering only the global indicator of general health hides associations between WFH and mental complaints in particular, underlining the usefulness of our approach to examine physical and mental health separately.

## Discussion and conclusion

With the recent global shift to WFH following the COVID-19 outbreak, many employers and employees are negotiating new WFH agreements. It is therefore increasingly important to identify potential benefits and drawbacks of WFH in terms of employees’ health outcomes, as health conditions can have great costs to society and the health care system and may negatively affect both work performance and quality of life.

Using large-scale survey data representative of employees in Germany, the current study contributed to the literature on the relationship between WFH and health outcomes in two main ways. First, distinguishing patterns of use and non-use of WFH, the study provided a precise empirical conceptualization of WFH. Previous research tended to look at WFH in a dichotomous way (use vs. non-use), which can produce misleading conclusions because it does not take into account different motives for non-use and use of WFH and different WFH arrangements (25). We distinguished between informal overtime at home and recognized telework, and in addition, considered the extent of telework. We also distinguished between employees who do not use WFH by choice (voluntary non-WFH users) or due to lack of permission from the employer (employer-directed non-WFH users). Second, using mental and physical health complaints instead of a global indicator of general health, we provided a more nuanced understanding of the positive and negative associations with WFH.

The empirical results of the study allow us to draw the following conclusions. The distinction between informal overtime and telework is important when assessing the relationship between WFH and employees’ health. Informal overtime at home is associated with various demands (e.g., time pressure, high workload, work-to-family conflict), and, thus, reflected in poorer mental health [e.g., (24, 27, 30, 40)]. By contrast, telework is perceived positively by most employees and has a greater potential to act as a resource (e.g., greater autonomy and flexibility, more time resources due to less commuting), so that employees who make use of it can be mentally and physically healthier than those who cannot draw on this resource [e.g., (2, 9, 29, 55)].

However, our study also shows that within the group of teleworkers, the health benefits may vary by the extent of telework. Precisely, employees with small telework extents reported significantly fewer mental health complaints, whereas those teleworking more than 20% of their time did not differ in their mental health outcomes to employer-directed non-WFH users. This finding suggests that, as the telework extent increases, the potential drawbacks of this work arrangement—such as a deterioration in relationships with colleagues and/or an increasing blurring of the boundaries between work and private life—increasingly offset its benefits, such as greater flexibility and less commuting.

Furthermore, while our results indicate that informal overtime at home bears risks for employees’ mental health, it should be noted that we also found unpaid overtime in the office to be associated with more mental complaints, and the estimated effects were actually larger for this group (see Supplementary Table S2). Our judgment about whether WFH in order to serve informal overtime is bad (or maybe even good) for employees’ health thus depends on whether the total workload is fixed or not: If WFH causes employees to do more overtime than they would do if they only worked on-site, we would conclude that informal overtime at home is disadvantageous. If, by contrast, the number of overtime hours that have to be served remains the same regardless of workplace, doing the overtime at home rather than in the workplace may still be preferable, because employees can do it on their own terms.

Lastly, our results showed that voluntary non-WFH users reported fewer mental and fewer physical complaints compared to employer-directed non-WFH users. This supports Koh et al. (11), who point out that ignoring the difference between self-decided non-use of WFH and lack of employer permission may introduce bias in the analysis.

We acknowledge that our study has limitations. First, we use cross-sectional data, so the reported relationships between WFH and health should be understood as associations. However, we argue that we have accounted for much of the heterogeneity that could introduce potential confounding bias into the analyses, as we were able to (i) decompose WFH into its various forms of use and non-use, and (ii) control for a very rich set of individual, job, firm, and regional variables that can be considered as determinants of both WFH and health. Nevertheless, panel data would be desirable in future analyses to account for possible health selection of teleworkers. Second, as there were relatively few employees in the data who worked a medium proportion of hours from home, it was not possible to further differentiate the relatively broad middle category (21–80% of total weekly working time). It can be expected that the arrangement of working 2–3 days per week at home will become more common post-COVID [e.g., (56)]. However, data collected after the pandemic, and containing the type of questions we need for our analysis, is not (yet) available. Future research should thus focus on how these WFH arrangements relate to health outcomes. A comprehensive look at the pre-pandemic and post-pandemic periods will provide a more complete picture of the association between WFH and health complaints.

In terms of policy and practice, our findings suggest that WFH can be beneficial for employees’ health and should therefore be enabled by employers. However, care should be taken to ensure that employees benefit from teleworking even when using it extensively. Studies show that the positive effects of telework depend, e.g., on an employee’s ability to reorganize the boundaries between work and personal life, as well as on organizational culture [e.g., (57–59)]. This suggests that telework must be designed in a way that promotes its benefits, such as time flexibility and autonomy, while limiting its potential risks, such as social isolation, the blurring of work and private life or more (unrecognized) overtime. This requires appropriate regulations and a supportive corporate culture. How WFH arrangements are organized and supported by the employer is important, and companies should develop and implement appropriate policies. For example, they could provide trainings for employees on skills for dealing with flexible working in terms of time and place, and for supervisors on skills for managing employees from a distance. There should also be clear rules regarding, for example, the availability of employees outside of normal office hours. In addition, employers should consider carrying out a risk assessment of the home workplace, both in terms of physical (ergonomics, etc.) and mental health.

## Data Availability

Publicly available datasets were analyzed in this study. This data can be found at: Research Data Centre of the Federal Institute for Vocational Education and Training (BIBB) (https://doi.org/10.7803/501.18.1.1.10).

## Footnotes

^1^In Germany, the vast majority of the workforce – around two thirds – commute to work by car, so physical activity on the way to work rarely matters, see (60).

^2^In our sample, 320 employees did not indicate whether or not their overtime in the company was recognised by the employer. They are included in our analysis as “no information on recognition of overtime.”

^3^In order to offer deeper insights into which individual complaints of the two indices are related to WFH specifically, we estimated separate logit models predicting the likelihood of reporting each of the individual complaints (see Supplementary Tables S1, S2.
