# Supplementary material for: Working from home and health complaints: on the difference between telework and informal overtime at home
Source: Front Public Health. 2025 Feb 18;13:1465617. doi: 10.3389/fpubh.2025.1465617 (PMC11878154; doi:10.3389/fpubh.2025.1465617)
Supplement: Supplementary file 1 [file Data_Sheet_1.docx]

Supplementary Material

**Working from home and health complaints: On the difference between telework and informal overtime at home**

**Table A1.** **Association between** **WFH and single mental complaints, estimates from logit regression models**

|  | Nervousness, irritability | Insomnia | Fatigue, exhaustion | Dejection |
| --- | --- | --- | --- | --- |
| No WFH |  |  |  |  |
|  |  |  |  |  |
| Employer-directed non-WFH use | *Reference* | *Reference* | *Reference* | *Reference* |
|  |  |  |  |  |
| Voluntary non-WFH use | -0.285*** | -0.380*** | -0.298*** | -0.460*** |
|  | (0.077) | (0.089) | (0.058) | (0.080) |
| WFH |  |  |  |  |
|  |  |  |  |  |
| Informal overtime at home | 0.351*** | 0.412*** | 0.233** | 0.185 |
|  | (0.073) | (0.096) | (0.086) | (0.112) |
| Telework |  |  |  |  |
|  |  |  |  |  |
| < 20 %, irregular | -0.011 | -0.101 | -0.127* | -0.293*** |
|  | (0.061) | (0.060) | (0.058) | (0.062) |
| 21–80 % | 0.174 | 0.105 | 0.015 | -0.198 |
|  | (0.105) | (0.092) | (0.078) | (0.101) |
| > 80 %, always | 0.219* | 0.127 | 0.070 | -0.142 |
|  | (0.088) | (0.125) | (0.160) | (0.097) |
| Constant | -1.975*** | -2.350*** | -1.286*** | -2.054*** |
|  | (0.230) | (0.223) | (0.201) | (0.211) |
| Pseudo R² | 0.029 | 0.046 | 0.037 | 0.052 |
| Observations | 10,365 | 10,365 | 10,365 | 10,365 |

Logit coefficients with cluster-robust standard errors in parentheses, * p<0.05, ** p<0.01, *** p<0.001.

Notes: Control variables are included but not displayed. Estimates for the full models are available upon request.

**Table A2.** **Association between** **WFH and single physical complaints, estimates from logit regression models**

|  | Back | Neck, Shoulders | Arms | Hands | Hips | Legs | Feet | Knees |
| --- | --- | --- | --- | --- | --- | --- | --- | --- |
| No WFH |  |  |  |  |  |  |  |  |
|  |  |  |  |  |  |  |  |  |
| Employer-directed non-WFH use | *Reference* | *Reference* | *Reference* | *Reference* | *Reference* | *Reference* | *Reference* | *Reference* |
|  |  |  |  |  |  |  |  |  |
| Voluntary non-WFH use | -0.188** | -0.228** | -0.232* | -0.174 | -0.228* | -0.403*** | -0.438*** | -0.210* |
|  | (0.067) | (0.077) | (0.091) | (0.093) | (0.096) | (0.104) | (0.112) | (0.093) |
| WFH |  |  |  |  |  |  |  |  |
|  |  |  |  |  |  |  |  |  |
| Informal overtime at home | -0.004 | 0.048 | -0.316** | -0.450*** | -0.071 | 0.012 | -0.093 | 0.111 |
|  | (0.085) | (0.107) | (0.117) | (0.132) | (0.112) | (0.106) | (0.105) | (0.091) |
| Telework |  |  |  |  |  |  |  |  |
|  |  |  |  |  |  |  |  |  |
| < 20 %, irregular | -0.083 | -0.089 | -0.362*** | -0.177 | -0.392*** | -0.407*** | -0.264* | -0.170* |
|  | (0.068) | (0.073) | (0.079) | (0.093) | (0.083) | (0.101) | (0.113) | (0.083) |
| 21–80 % | 0.029 | -0.002 | -0.228 | -0.187* | -0.065 | -0.260 | -0.396** | -0.070 |
|  | (0.076) | (0.110) | (0.153) | (0.089) | (0.158) | (0.147) | (0.140) | (0.124) |
| > 80 %, always | -0.062 | -0.119 | -0.440** | -0.378** | -0.515*** | -0.336* | -0.304 | -0.031 |
|  | (0.096) | (0.107) | (0.155) | (0.133) | (0.090) | (0.152) | (0.162) | (0.109) |
| Constant | -1.550*** | -1.423*** | -3.128*** | -3.292*** | -4.038*** | -3.589*** | -2.841*** | -3.032*** |
|  | (0.243) | (0.154) | (0.291) | (0.275) | (0.302) | (0.355) | (0.272) | (0.160) |
| Pseudo R² | 0.049 | 0.057 | 0.099 | 0.094 | 0.089 | 0.101 | 0.130 | 0.127 |
| Observations | 10,365 | 10,365 | 10,365 | 10,365 | 10,365 | 10,365 | 10,365 | 10,365 |

Logit coefficients with cluster-robust standard errors in parentheses, * p<0.05, ** p<0.01, *** p<0.001.

Notes: Control variables are included but not displayed. Estimates for the full models are available upon request.

.

**Figure A1. Average probabilities of mental and physical complaints by WFH (with 95 % confidence interval)**

Notes: Estimates from models M3a and M3b.

.

**Table A3.** **Robustness checks**

|  | **RC 1.1** | | **RC 1.2** | | **RC 2** | | **RC 3** |
| --- | --- | --- | --- | --- | --- | --- | --- |
|  | **Mental complaints** | **Physical complaints** | **Mental complaints** | **Physical complaints** | **Mental complaints** | **Physical complaints** | **General health** |
|  |  |  |  |  |  |  |  |
| No WFH |  |  |  |  |  |  |  |
| No WFH, job tasks | -5.718*** | -1.782** |  |  |  |  |  |
|  | (0.696) | (0.654) |  |  |  |  |  |
| Employer-directed non-WFH use | *Reference* | *Reference* | *Reference* | *Reference* | *Reference* | *Reference* | *Reference* |
|  |  |  |  |  |  |  |  |
| Voluntary non-WFH use | -6.709*** | -3.376*** |  |  | -6.747*** | -3.334*** | 1.493*** |
|  | (1.010) | (0.749) |  |  | (1.026) | (0.773) | (0.114) |
| WFH |  |  |  |  |  |  |  |
|  |  |  |  |  |  |  |  |
| Informal overtime at home | 5.947*** | -0.868 | 8.428*** | 0.123 |  |  | 1.122 |
|  | (1.172) | (0.580) | (1.172) | (0.510) |  |  | (0.068) |
| < 5 % |  |  |  |  | 6.518*** | -0.878 |  |
|  |  |  |  |  | (1.639) | (0.783) |  |
| 5-15 % |  |  |  |  | 5.280** | -0.613 |  |
|  |  |  |  |  | (1.614) | (0.857) |  |
| > 15 % |  |  |  |  | 6.993* | -1.656 |  |
|  |  |  |  |  | (3.016) | (1.231) |  |
| Telework |  |  |  |  |  |  |  |
|  |  |  |  |  |  |  |  |
| < 20 %, irregular | -2.720** | -2.324** | -0.272 | -1.497* | -2.412** | -2.558*** | 1.339*** |
|  | (0.849) | (0.695) | (0.698) | (0.606) | (0.865) | (0.650) | (0.081) |
| 21–80 % | 0.313 | -1.202 | 2.864* | -0.487 | 0.684 | -1.573 | 1.292** |
|  | (1.408) | (1.031) | (1.242) | (0.894) | (1.392) | (0.969) | (0.109) |
| > 80 %, always | 1.390 | -3.308** | 3.823* | -2.337** | 1.635 | -3.437** | 1.098 |
|  | (1.832) | (0.975) | (1.896) | (0.727) | (1.987) | (0.902) | (0.083) |
| Constant | 10.738*** | 4.640** | 8.043** | 3.418 | 10.66*** | 4.660* |  |
|  | (1.777) | (1.607) | (2.667) | (2.355) | (2.657) | (2.241) |  |
| R² | 0.070 | 0.211 | 0.069 | 0.187 | 0.073 | 0.189 | 0.060 (Pseudo R²) |
| Observations | 15,551 | 15,551 | 10,365 | 10,365 | 10,365 | 10,365 | 10,351 |

B-coefficients (for RC 1.1, 1.2, and 2) and Odds ratios (for RC 3) with cluster-robust standard errors in parentheses, * p<0.05, ** p<0.01, *** p<0.001.

Notes: Control variables are included but not displayed. Estimates for the full models are available upon request.
